# Strategy of Nematophagous Fungi in Determining the Activity of Plant Parasitic Nematodes and Their Prospective Role in Sustainable Agriculture

**DOI:** 10.3389/ffunb.2022.863198

**Published:** 2022-05-16

**Authors:** Laith Khalil Tawfeeq Al-Ani, Filippe Elias de Freitas Soares, Ashutosh Sharma, Sergio de los Santos-Villalobos, Ana Victoria Valdivia-Padilla, Liliana Aguilar-Marcelino

**Affiliations:** ^1^Department of Plant Protection, College of Agriculture, University of Baghdad, Baghdad, Iraq; ^2^School of Biology Science, Universiti Sains Malaysia, Kubang Kerian, Malaysia; ^3^Department of Chemistry, Universidade Federal de Lavras, Lavras, Brazil; ^4^Tecnologico de Monterrey, School of Engineering and Sciences, Centre of Bioengineering, Queretaro, Mexico; ^5^Instituto Tecnológico de Sonora, Ciudad Obregón, México; ^6^Centro Nacional de Investigación Disciplinaria en Salud Animal e Inocuidad, INIFAP, Jiutepec, Mexico

**Keywords:** *Trichoderma*, *Pleurotus*, melanin, nematode-trapping fungi, non-pathogenic, *Fusarium*

## Abstract

In this review, we supply a framework for the importance of nematophagous fungi (nematophagous fungi [NF]) and their role in agricultural ecosystems. We characterize the taxonomy, diversity, ecology, and type of NF, depending on their interaction with plant-parasitic nematodes (PPNs). We described potential mechanisms of NF in the control of PPNs, the efficiency and methods of utilization, and the use of nematicides in sustainable agriculture. We explain the utilization of NF in nanotechnology as a new approach. NF are significant in the soil for having the effective potential for use in sustainable agriculture. These types of fungi belong to wide taxa groups, such as Ascomycota, Basidiomycota, and other groups. Diverse NF are available in different kinds of soil, especially in soils that contain high densities of nematodes. There is a relationship between the environment of nematodes and NF. NF can be divided into two types according to the mechanisms that affect nematodes. These types are divided into direct or indirect effects. The direct effects include the following: ectoparasites, endoparasites, cyst, or egg parasites producing toxins, and attack tools as special devices. However, the indirect effect comprises two groups: paralyzing toxins and the effect on the life cycle of nematodes. We explained the molecular mechanisms for determining the suitable conditions in brief and clarified the potential for increasing the efficacy of NF to highly impact sustainable agriculture in two ways: directly and indirectly.

## Introduction

Plant–parasitic nematodes (PPNs) in soil can be prey for several pathogens, such as viruses, -bacteria, and fungi. Many groups of these pathogens can be utilized in the biological control of nematodes (Rilrron, 1977).

However, using new control methods instead of synthetic chemicals is necessary to improve sustainable agriculture (Aguilar-Marcelino et al., 2020a,b, 2021; Al-Ani et al., 2020a, 2021a,b; Sharma et al., 2020; Singh et al., 2021). Many plant pathogens, such as *Alternaria* spp. (Garganese et al., 2018), *Aspergillus* spp. (Attitalla et al., 2010a,b), and *Fusarium* spp. (Mohammed and Al-Ani, 2021), caused a high reduction in plant growth and yield. The utilization of natural factors showed high efficacy in conferring protection for plants from plant pathogens and pests. Plant growth promoting rhizobacteria (PGPR) biocontrol agents showed the ability to control several plant pathogens (Al-Ani, 2006, 2017a, 2018a,b; Al-Ani and Al-Ani, 2011; Mohammed et al., 2011, 2014; Al-Ani et al., 2020b; Soumare et al., 2021). *Trichoderma* is a more interesting genus used in biological control that has shown efficacy in the management of several plant diseases (Al-Ani et al., 2013a, 2018; Al-Ani and Albaayit, 2018a,b; Al-Ani, 2019a,b; Al-Ani and Mohammed, 2020) and has been detected as act endophyte in plants (Al-Ani, 2019c,d,e).

Non-pathogenic fungi and entomopathogenic fungi controlling plant disease and pests (Al-Ani et al., 2018; Al-Ani, 2019c,f; Gupta et al., 2022), have the ability to manage some plant diseases and play a role in sustainable agriculture by enhancing plant vigor (Al-Ani and Salleh, 2010; Al-Ani et al., 2013b; Al-Ani, 2017b, 2019d,e; Al-Ani and Furtado, 2020; Kisaakye et al., 2022). In addition, many natural compounds and plant extracts are helpful in controlling plant diseases and pests (Al-Ani et al., 2012; Mohammed et al., 2012, 2013; Adetunji et al., 2019; Garganese et al., 2019; Jatoi et al., 2020). NF are used in the control of parasitic nematodes as bionematicides by parasitism, and produce toxins to kill nematodes, as well as induce defense and resistance mechanisms in plants against parasite nematodes (Abd-Elgawad and Askary, 2018; Sarker et al., 2020; Comans-Pérez et al., 2021; Girardi et al., 2022). NF as biological control factors are the best method to use in sustainable agriculture. NF belong to most groups of fungal taxa, such as Oomycota, Zygomycota, Ascomycota, Pleurotaceae (Basidiomycota), and Chytridiomycetes (Gams and Zare, 2003; Wijayawardene et al., 2020).

Many NF have not yet been discovered, and around 6,000–8,000 species are waiting for identification (Li et al., 2000; McInnes, 2003; Yang et al., 2012). NF inhabit the soil and rhizosphere (Liu et al., 2009) and have been detected in several soils but not in soil from extreme environments, such as high salt concentrations and high temperatures (Yang et al., 2020). NF are divided into five types: (A) nematode-trapping fungi, (B) endoparasitic fungi, (C) fungi that secrete toxins affecting nematodes, (D) egg-parasitic fungi, and fungi that induce resistance and defenses in plants, which then influence the activity of or kill PPNs (Swe et al., 2011; Maia Filho et al., 2013).

The secondary metabolites of some NF shown high efficacy in controlling parasitic nematodes (Castañeda-Ramírez et al., 2020; Seong et al., 2021). In the present review, we discuss the strategy of NF in the control of plant-parasitic nematodes and their activity in sustainable agriculture.

## Mechanism of Nematophagous Fungi to Control PPNs

Soil NF are a heterologous group that acts as a natural enemy of parasitic nematodes. These fungi use nematode biomass as a source of carbon, nitrogen, and other important elements (Siddiqui and Mahmood, 1996); some of them are obligate parasites of nematodes, but the majority are facultative saprophytes (Lopez-Llorca et al., 2007).

These NF have been studied for their use as a biological control against PPNs. Biological control of phytonematodes can be defined as the decrease in nematode populations by the action of living organisms other than those naturally found in the host plant by the manipulation of the environment or by the introduction of antagonist organisms (Kim, 2015). To this day, more than 200 species of taxonomically diverse fungi have shown the ability to attack living nematodes in their different stages: juvenile, adults, and eggs (Nordbring-Hertz et al., 2006).

**Figure 1:**
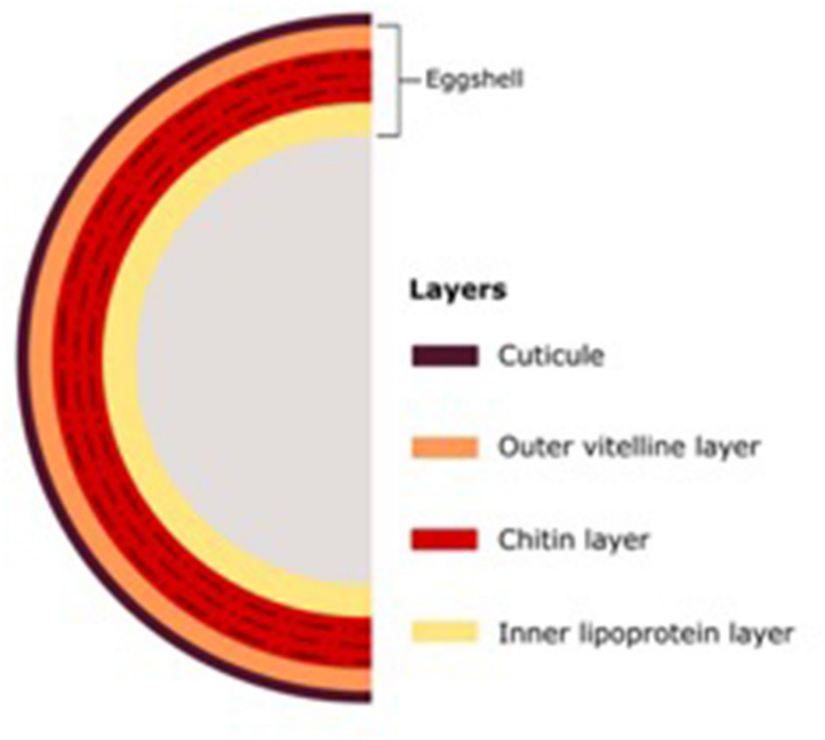
The structure of eggshell layers.

**Figure 2:**
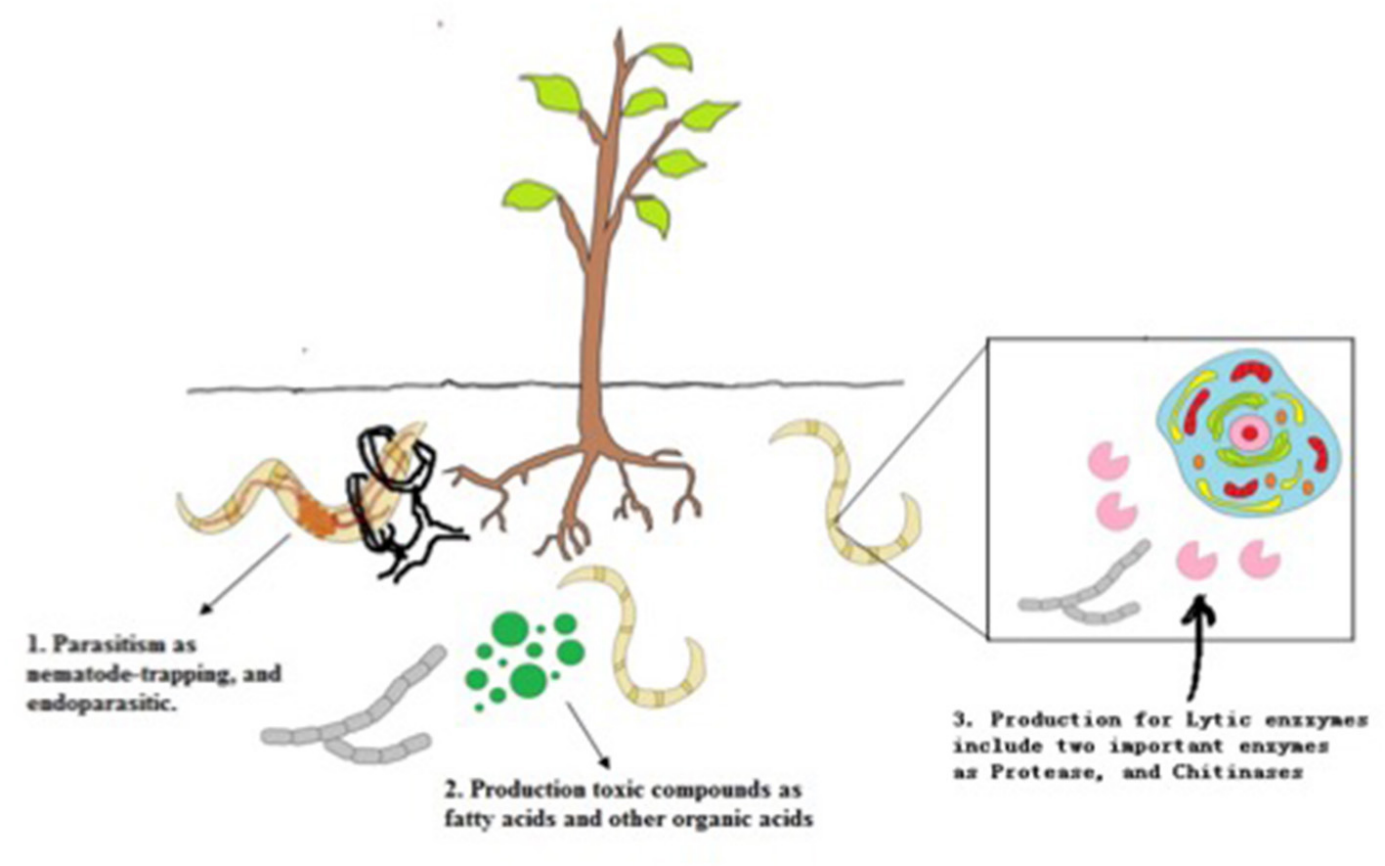
The types of mechanisms used by NF to infect nematodes.

The morphology of nematodes presents two different barriers against fungal infection. The first is the eggshell, which in root-knot and cyst nematodes consists of three layers: the outer vitelline composed mainly of proteins, the chitin layer, and the inner lipoprotein layer; and the second barrier, the cuticle (Figure 1). The thickness of these barriers varies considerably depending on the nematode genus (Morton et al., 2004). The types of mechanisms used by NF to infect nematodes can be divided into parasitism, toxic compounds, and enzymes (Figure 2).

### Parasitism

**Figure 3:**
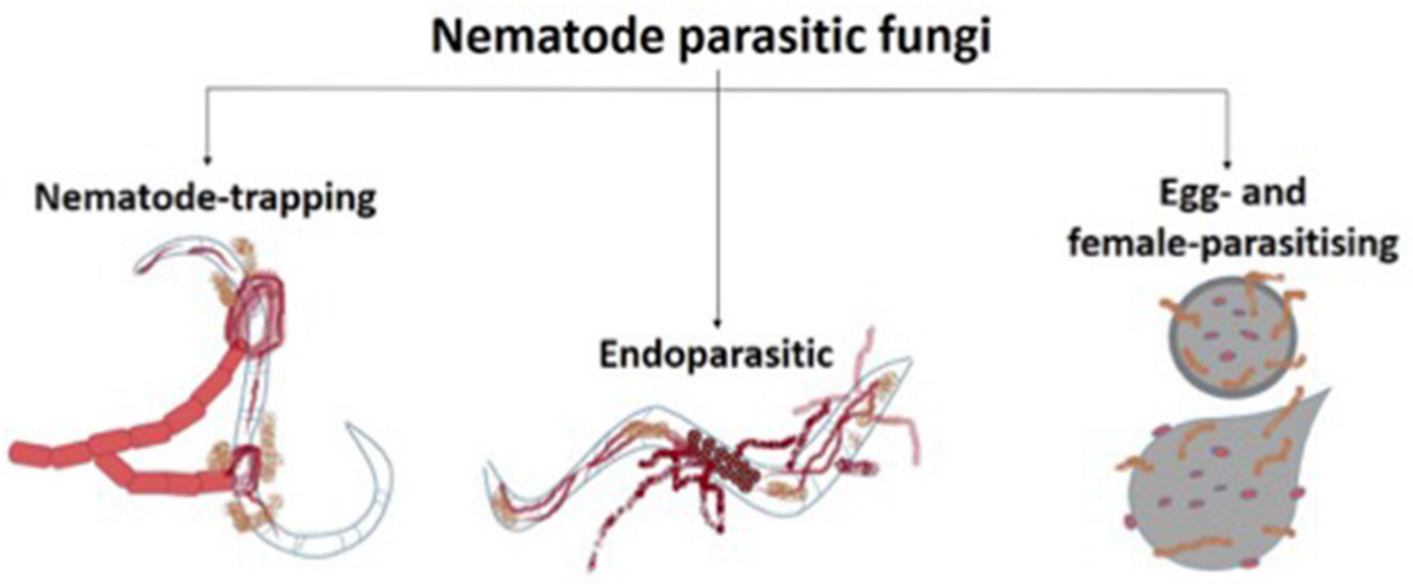
Types of nematode parasitic fungi.

Nematode parasitic fungi are those who live on or in their host organism and benefit from it by obtaining food. In this group, fungi can be divided into nematode-trapping, endoparasitic, and egg- and female-parasitising fungi (Figure 3; Abd-Elgawad and Askary, 2018).

Nematode-trapping fungi enter their parasitic stage by producing specialized structures for the capture of nematodes in their mycelium. These sessile structures depend on the species and strain of the fungus, and the environmental conditions in which it is found, such as biotic and abiotic conditions, living nematodes being the most important biotic factor, since they induce the formation of trapping structures by contact with the mycelium (Nordbring-Hertz et al., 2006). These structures act as two-dimensional or three-dimensional adhesive nets, adhesive knobs, or constrictor rings, so the fungi can invade the nematode and employ them as an additional source of food (Lopez-Llorca et al., 2007).

The traps formed by fungal mycelium act as a piercing mechanism, damaging the cuticle of the nematode. A penetration peg is formed, and the hyphae infect and grow throughout the interior of the nematode body. Finally, the hyphae project themselves through the exterior of the colonized nematode (Soares et al., 2018). The fungus *Arthrobotrys oligospora* is a nematode-trapping fungus that forms a specialized penetration tube to pierce the cuticle of the nematode and has been shown to have a significant impact on the control of the nematode *Meloidogyne javanica* in tomato cultivars under greenhouse conditions (Mostafanezhad et al., 2014).

Another group of NF is endoparasitic and does not form specialized structures to infect nematodes but rather produces spores (conidia or zoospores) to fulfill this function. Most of this group are obligate parasites of nematodes and carry out their complete vegetative stages within the nematode (Lopez-Llorca et al., 2007). The spores of these fungi infect the nematode when ingested, such as those produced by *Harposporium* spp., or by the adhesion of these spores to the host's cuticle, to later inject their content into the nematode, as in the case of *Drechmeria coniospora* and *Verticillium* spp. (Morton et al., 2004).

In the case of zoospore-producing fungi, such as *Pythium caudatum*, its spores are attracted to the nematode's secretions, causing them to swim toward it and encyst around the nematode's natural orifices, such as the mouth, anus, or vulva. Zoospores become immobile, germinate, and begin to form a hyphal penetration tube that enters through the body orifice into the nematode (Kim, 2015).

Endoparasitic fungi can also specialize in infecting eggs and female parasites when targeting the non-motile stage of the nematode, which is the egg; their hyphae grow toward nematode eggs and form appressoria, specialized flattened and enlarged hyphal tips that adhere to surfaces and facilitate penetration of the eggshell (Nordbring-Hertz et al., 2006). The infected eggs swell and buckle as penetration continues, and the fungi can digest their content, obtaining nutrients and energy to continue their growth (Kim, 2015). An example of this category for its use as a natural plant–parasitic nematode control is the fungus *Trichoderma harzianum*, which showed a significant reduction in the population and egg masses of the root-knot nematode *M. incognita* in tomato plants (Feyisa and Lencho, 2015).

### Toxic Compounds

Some species of NF produce certain chemical compounds that are toxic to nematodes. These compounds lead to nematode paralysis, and subsequently, the fungus consumes them. In some cases, nematode head shrinkage is seen as a side effect of the action of toxins (Satou et al., 2008). These NF are classified as toxin-producing fungi. However, most studies regarding NF have focused on the predatory and endoparasitic fungi (Soares et al., 2018).

Most toxin-producing fungi are basidiomycetes. In this context, several species of edible mushrooms from the *Pleurotus* genus produce toxins with nematotoxic activity (Kwok et al., 1992; Nordbring-Hertz et al., 1995; Satou et al., 2008). For example, *P. ostreatus* produces trans-2-decenoic acid, a compound derived from linoleic acid that is toxic to nematodes, insects, and other fungi (Kwok et al., 1992). However, it should be noted that basidiomycetes do not only produce these types of toxins, but also there are some fungi that produce compounds which are toxic to nematodes but are not nematophagous, i.e., do not consume the nematode (Soares et al., 2018).

The chemical characteristics of these compounds are also quite diverse, including simple fatty acids and other organic acids, such as pyrones, lactones, benzoquinones, anthraquinones, furans, alkaloids, cyclodepsipeptides, and peptaibiotics, and hybrid structures, such as lactam-bearing macrolactones (Degenkolb and Vilcinskas, 2016a). Toxin-producing fungi and their metabolites have been brilliantly reviewed in the works of Degenkolb and Vilcinskas, 2016a,b.

### Enzymes

All five groups of NF (nematode-trapping/predators, opportunistic or ovicidal, endoparasites, toxin-producing fungi, and producers of special attack devices) share necessary weapons for the infection and digestion of nematodes, namely enzymes (Braga and de Araújo, 2014; Soares et al., 2018). These macromolecules have the biological activity of catalyzing reactions. Thus, the reactions are accelerated through the action of enzymes.

Nematodes have physical barriers in their constitution that protect them from the actions of natural predators. The cuticle of juvenile PPNs is one of these barriers (Lee, 1967; Ekino et al., 2017). In its composition, there is an abundance of proteins. To overcome this barrier, NF have mechanical and enzymatic approaches. Regarding enzymes, proteases (EC 3.4) such as lkaline serine protease, and neutral serine protease are the main macromolecules involved in cuticle digestion. The poteases enzymes catalyze the hydrolysis of the peptide bonds of cuticular proteins (Liang et al., 2010). lkaline serine protease produced by *Lecanicillium psalliotae* (syn. *Verticillium psalliotae*) caused degradation of cuticles within hours and immobilized the nematode *P. redivivus* (Yang et al., 2005). *Arthrobotrys oligospora* produced neutral serine protease playing a role in pathogenicity against nematode (Zhao et al., 2004). *Arthrobotrys oligospora* is a useful in controlling *Haemonchus contortus* and *Caenorhabditis elegans in vitro* by producing serine proteases (Junwei et al., 2013; Yang et al., 2022). Therefore, their role is crucial in the fungus infection process. However, the eggs of PPNs have shells rich in chitin and protein. Chitinases (EC 3.2.1.14) such as Endochitinase, and exochitinase (Tikhonov et al., 2002), are enzymes that catalyze the hydrolysis of glycosidic bonds between the N-acetylglucosamine groups of chitins. Therefore, the fundamental fungal enzymes in the process of infection and digestion of this shell are chitinases (Khan et al., 2004). NF *Monacrosporium thaumasium* produced chitinases (Extracellular) that showed nematicidal action against nematode *Panagrellus redivivus* (Soares et al., 2014).

In addition to acting in harmony with the mechanical mechanisms of infection and digestion of NF, enzymes have proven nematicidal action when used alone, without the presence of fungi (Soares et al., 2012; Braga et al., 2015). Thus, this opens up the possibility of new approaches for the control of PPNs.

### Special Attack Devices

Some types of nematophagous produce a special device using in attacking against nematodes (Soares et al., 2018). The device is similar to tools used by nematophagous fungi to cause harm to the cuticles of nematodes and then complete the attack on the nematode. The shape of devices? is differing such as sword, racket with thorns, and spear (Soares et al., 2018). We can write the steps of using these devices in attacking into four points (Luo et al., 2004, 2006, 2007), as follows:

(A)The growth of hyphae is being toward of nematode, then press it.
(B) Formation the penetration peg is using to penetrates the nematodecuticle.
(C) Then, the infect with nematophagous fungi will complete for body of nematode by hyphae.

## Efficacy of NF In Sustainable Agriculture

Nematophagous fungi are more helpful for the biological control of PPNs compared to other organisms, such as bacteria and viruses. These NF are available in the environment of prey nematodes. The importance of NF can be divided into two types: direct and indirect effects. Direct effects indicate the capability of NF to directly affect nematodes by parasitising adults, juveniles, and eggs, as well as producing toxins or secondary metabolites causing nematode immobility. Many NF use the mechanisms mentioned previously. For indirect effects, this mechanism can happen by inducing both plant defenses (Lopez-Llorca et al., 2010), and plant resistance in monocotyledon and dicotyledon (Bordallo et al., 2002), that affect the activity of PPNs, such as laying eggs, hatching eggs, completing the life cycle from juveniles to adults, and immobilization. Two nematophagous fungi *Arthrobotrys oligospora* and *Verticillium chlamydosporium* colonized the plants both of barley and tomato (Bordallo et al., 2002). Endophytic *Fusarium oxysporum* showed the capability to reduce the population of PPNs *Meloidogyne* sp. after it was used to treat bananas (Waweru et al., 2014). Some biocontrol fungi, such as *Trichoderma*, can be useful in reducing the pathogenicity activity of nematodes by affecting juveniles (an important stage for causing plant disease) and eggs. *Trichoderma asperellum* T-16 reduced the densities of juveniles (second-stage J2s) in roots by about 80%, but *T*. *brevicompactum* T-3 could suppress the production of eggs by around 86% (Affokpon et al., 2011).

## Methods Utilizing Nematophagous Fungi In Sustainable Agriculture

Plant–parasitic nematodes are a major cause of losses in world agriculture, resulting in yield and monetary losses. Nematodes can grow undetected by the farmer due to their microscopic size, underground habitat, and non-specific plant infection symptoms (Kim, 2015). Much damage to crops goes unreported or is often classified with other causes, such as fungal attacks, hydric stress, nutritional deficiencies, or other soil factors (Abd-Elgawad and Askary, 2018).

Chemical nematocides are the most traditional option for the management of PPNs, but their application is increasingly being re-evaluated because of their multiple environmental and health hazards, in addition to their low availability and high cost (Degenkolb and Vilcinskas, 2016a,b). Another major disadvantage of the frequent use of these chemicals is that they can result in the generation of resistant nematode races, which can make their control even more difficult (Abd-Elgawad and Askary, 2018).

Because NF can actively feed on and are the natural antagonist of PPNs. This trend includes the study of these fungi in their application form, shelf life, culture, mass production, effectiveness with other biotic and abiotic factors, and crop management techniques (Kim, 2015).

**Figure 4:**
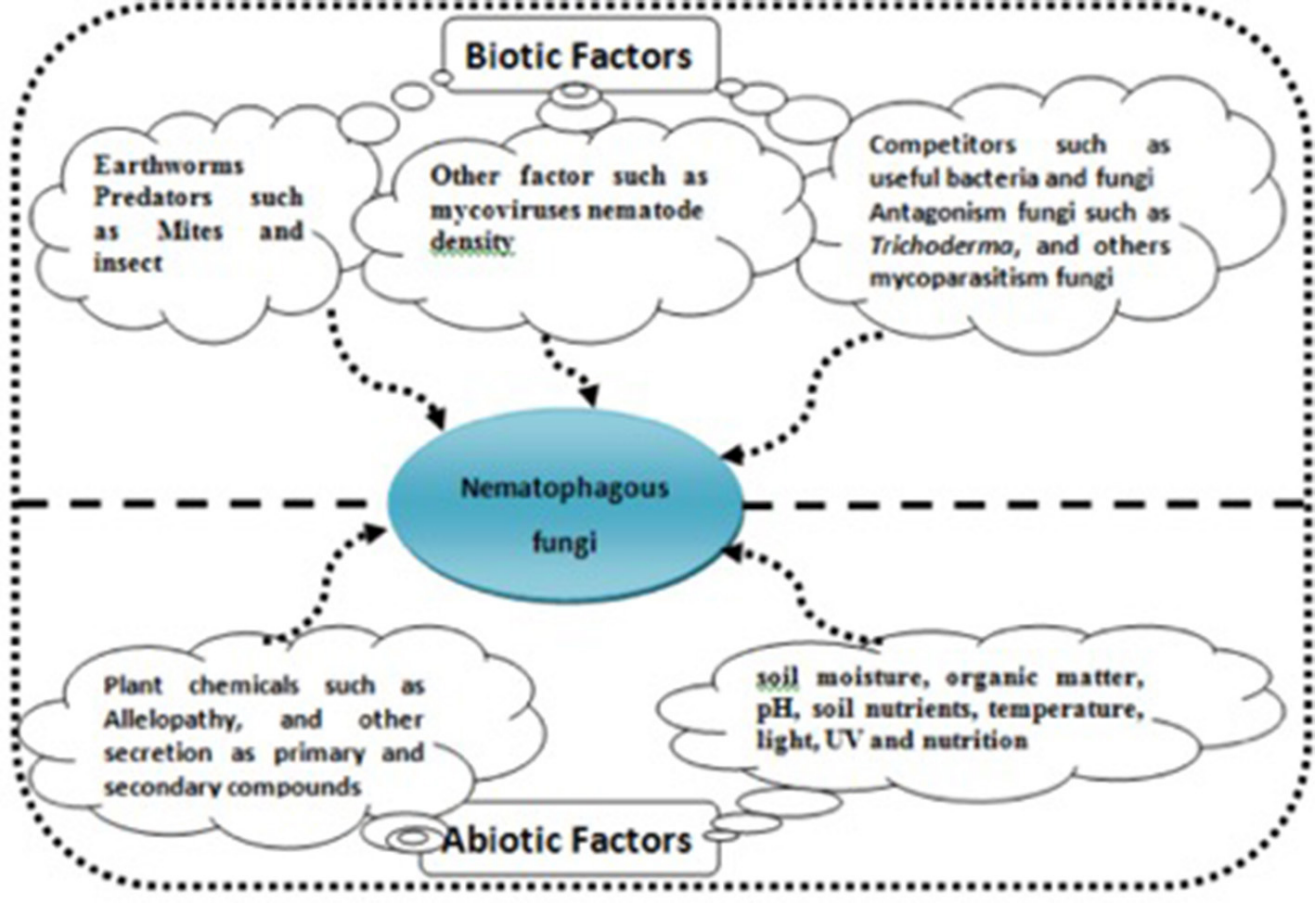
Thi figure showed biotic and abiotic factors that affect the biocontrol agent nematophagous fungi.

Bioproducts with these NF in their formulation present several benefits for more sustainable agriculture against chemical nematicides, such as easy application, environment safety, do not affect the soil biota, and do not leave residues in harvested products. However, as a living system, there are several considerations to note when developing a commercial bionematicidal product. To accomplish effective and reproducible biological control, this study is important for the determination of both the biotic and abiotic factors that affect the biocontrol agent and the target parasite (Figure 4). The fungi to be used must be able to grow well in the field to be treated, considering the influence of chemical, physical, and other biological factors in the soil that act like fungistatic compounds, in addition to the fact that these factors can be difficult to predict due to crop rotations (Yang et al., 2007).

Therefore, the use of new technology has been incorporated into biopesticide production. One example is the use of real-time quantitative PCR technology to quantify and track the biocontrol agent after it has been applied to the soil. Similarly, genetic modification of biocontrol agents can improve their efficacy by achieving overexpression of genes involved in pathogenicity or nematocidal activity (Zhang et al., 2020a,b). Genetic modification approaches are utilized for enhancing the virulence, aggression against nematodes, UV protectants, expression of heat shock factors, immune modulators, and cuticle degrading enzymes.

There are two general techniques for using NF as a biological control agent. One technique is the addition of large amounts of fungi to the soil or as an endophytic organism of the plant by coating seeds to favor the colonization of its rhizosphere. The fungus can establish itself before the nematodes are attracted to the plant roots (Nordbring-Hertz et al., 2006). The second technique is to improve the bionematicide formulation with the complementary use of biocontrol agents nematophagous fungus the improve the growth, adaptability, and efficiency of NF as an integrated pest management technique (Yang et al., 2007). In this situation, the integrated use of a fungus as a biocontrol agent in addition to a lower dose of chemical pesticide or plant product can give effective results if the components are compatible (Kim, 2015).

There have been various formulations to maximize the action of the biopesticide. A study of the fungus *T*. *viride* in combined application with the chemical nematicide carbofuran has shown an increase in plant height, root length, and decreased nematode *M*. *graminicola* population in rice crops (Pankaj et al., 2015).

Similarly, a formulation of the ovicidal fungus *P*. *lilacinus* added to the plant product neem cake shows egg parasitism of the nematode *N*. *incognita* and plant growth in tomatoes (Zaki and Maqbool, 1992). Other formulations include the use of the phytohormone abscisic acid (ABA), which enhances plant defense and increases the nematode-trapping capability of *D*. *stenobrocha*, a constricting ring-forming fungus (Xu et al., 2011). Different studies have also demonstrated an effective integrated pest management technique for the PPNs with bacteria *P*. *fluorescens* or a combination of several species of fungi (Abd-Elgawad and Askary, 2018).

## Conclusions

The interaction between fungi and nematodes is interesting, especially the use of NF as an alternative to synthetic chemicals that are used in the manufacture of nematocides. In recent years, techniques have been developed for genome sequencing and transcriptome analysis, as well as for analysis techniques for detecting chemicals at the nano level. We have a lot of information about the relationship between NF and nematodes that comprises enzymes and secondary metabolites as toxins. This antagonistic relationship depends on creating a special structure to capture the host and produce a toxin. Information on the interactions can be supplied through “-omics” data. The enhancement in the manufacture of bionematicides is depending on selecting aggressive isolates of NF by determining the virulence factors to a molecular level for isolates of NF. Efficiently obtaining bionematicides is a target and requirement for all researchers in the field of agriculture sustainability. Some information is still lacking, depending on the factor of pathogenicity.

Some enzymes, such as serine proteases, chitinases, and toxins, of NF are more interesting in the process of infection against parasitic nematodes through their role as virulence factors. The production of different enzymes in the process of infection of parasitic nematodes requires their penetration of diverse layers of cuticle and eggshell. The success of some strains of NF indicates differences in host preference. Some NF, such as the genus *Trichoderma*, play an important role in sustainable agriculture. Finally, we consider the high utilization of NF in the control of PPNs as an alternative to synthetic chemicals, and they may be more useful in sustainable agriculture, reducing harmful chemical residues in the ecosystem.

## Author Contributions

All authors listed have made a substantial, direct, and intellectual contribution to the work and approved it for publication.

## Funding

This present mini review article was partially financed by the Proyectos Fiscales Instituto Nacional de Agricultural and Livestock Forestry Research, INIFAP (project number: 139335341).

## Conflict of Interest

The authors declare that the research was conducted in the absence of any commercial or financial relationships that could be construed as a potential conflict of interest.

## Publisher's Note

All claims expressed in this article are solely those of the authors and do not necessarily represent those of their affiliated organizations, or those of the publisher, the editors and the reviewers. Any product that may be evaluated in this article, or claim that may be made by its manufacturer, is not guaranteed or endorsed by the publisher.
